# Supplementary figures and images for: Insights into the metagenomic and metabolomic compositions of the bacterial communities in Thai traditional fermented foods as well as the relationships between food nutrition and food microbiomes
Source: PeerJ. 2025 Jun 27;13:e19606. doi: 10.7717/peerj.19606 (PMC12208106; doi:10.7717/peerj.19606)

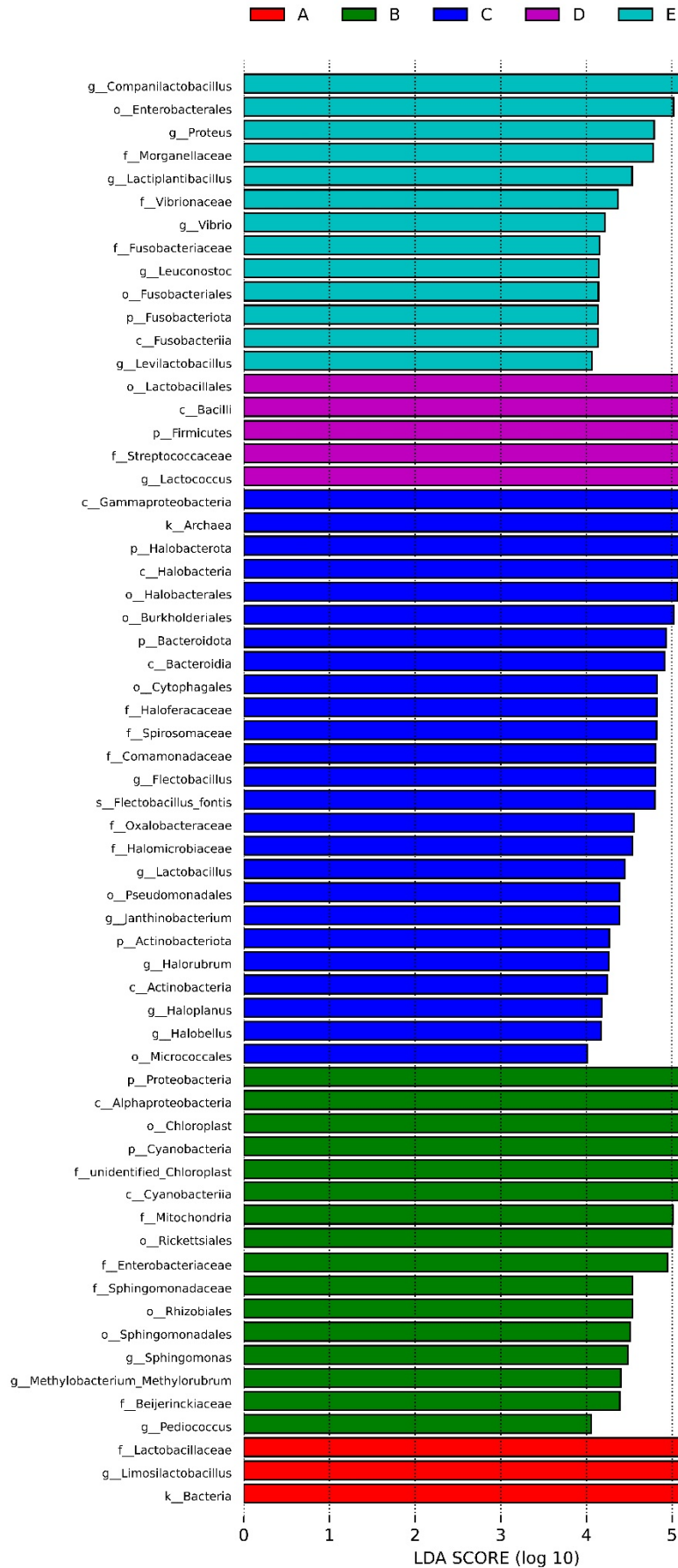

Supplement: Supplemental Information 1 — Sample codes (A-E) are as those in Table 1 footnote. [file peerj-13-19606-s001.pdf]

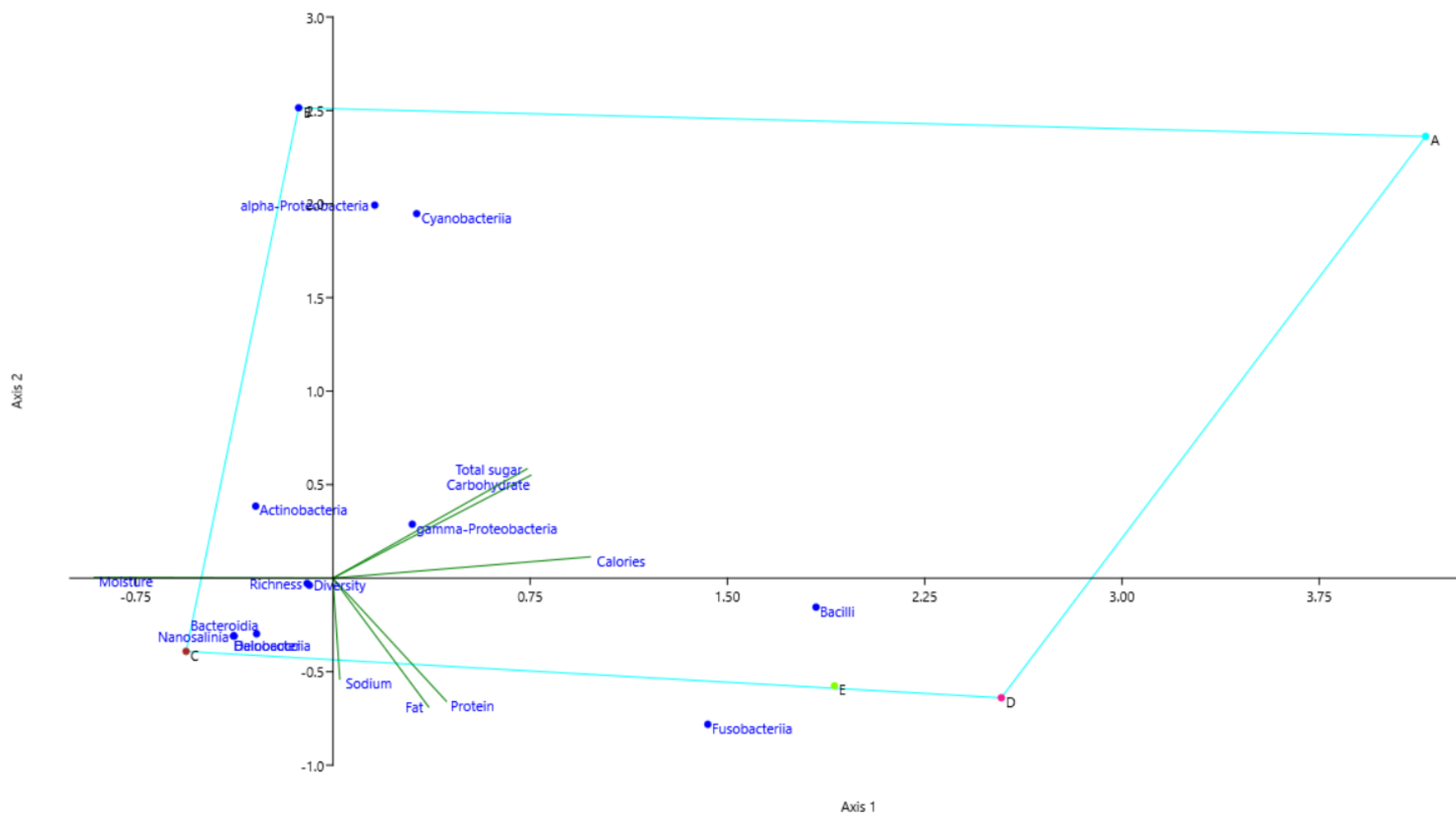

Supplement: Supplemental Information 2 — Food nutrition parameters influencing bacterial communities are represented by green lines whose lengths represent effect levels. [file peerj-13-19606-s002.pdf]

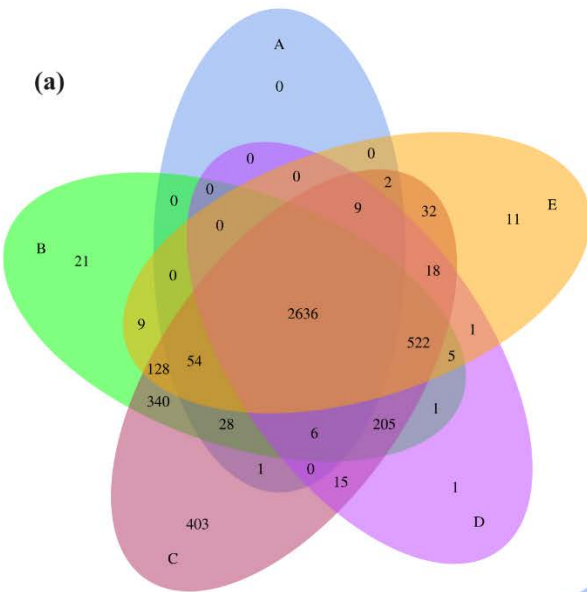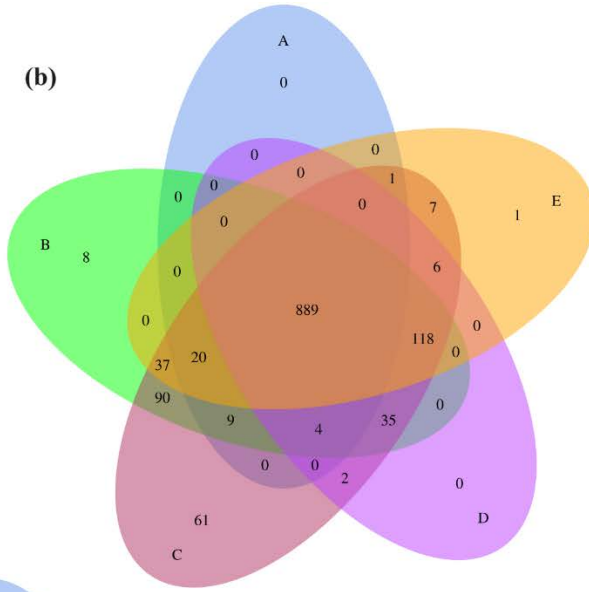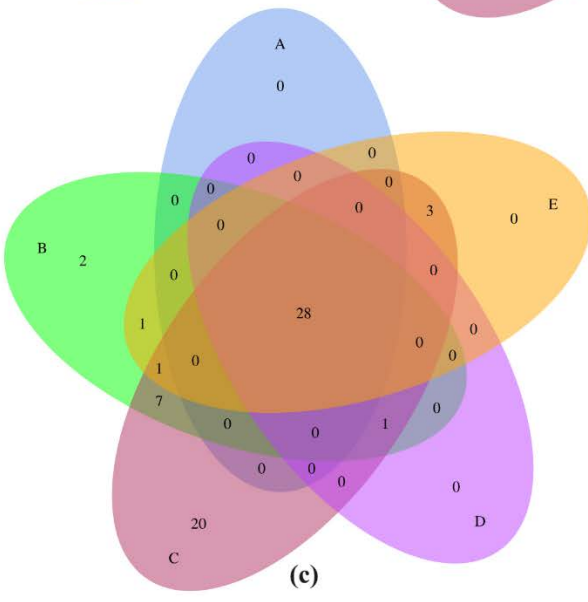

Supplement: Supplemental Information 3 — (A) Genes (B) Enzymes (C) Metabolic pathways. Sample codes (A-E) are as those in Table 1 footnote. [file peerj-13-19606-s003.pdf]
