# Supplementary material for: Insights into the metagenomic and metabolomic compositions of the bacterial communities in Thai traditional fermented foods as well as the relationships between food nutrition and food microbiomes
Source: PeerJ. 2025 Jun 27;13:e19606. doi: 10.7717/peerj.19606 (PMC12208106; doi:10.7717/peerj.19606)
Supplement: Supplemental Information 4 [file peerj-13-19606-s004.docx]

| Spearman's correlations | | | | | | | | |
| --- | --- | --- | --- | --- | --- | --- | --- | --- |
| Bacterial class | | Calories | Carbohydrate | Fat | Moisture | Protein | Total sugar | Sodium |
| Diversity (ASV number) | Correlation Coefficient | -.820^**^ | -0.364 | -.564^*^ | .810^**^ | -.517^*^ | -.700^**^ | 0.050 |
|  | Sig. | 0.000 | 0.183 | 0.028 | 0.000 | 0.048 | 0.004 | 0.859 |
| Richness | Correlation Coefficient | -.821^**^ | -0.360 | -.569^*^ | .804^**^ | -0.514 | -.694^**^ | 0.050 |
|  | Sig. | 0.000 | 0.188 | 0.027 | 0.000 | 0.050 | 0.004 | 0.860 |
| *Bacilli* | Correlation Coefficient | 0.493 | -0.113 | .912^**^ | -.514^*^ | .818^**^ | -0.005 | .536^*^ |
|  | Sig. | 0.062 | 0.689 | 0.000 | 0.050 | 0.000 | 0.986 | 0.040 |
| alpha-*Proteobacteria* | Correlation Coefficient | -0.414 | 0.045 | -.829^**^ | 0.468 | -.865^**^ | 0.235 | -.621^*^ |
|  | Sig. | 0.125 | 0.874 | 0.000 | 0.079 | 0.000 | 0.399 | 0.013 |
| gamma-*Proteobacteria* | Correlation Coefficient | -.774^**^ | -0.297 | -.592^*^ | .786^**^ | -0.472 | -.690^**^ | 0.091 |
|  | Sig. | 0.001 | 0.282 | 0.020 | 0.001 | 0.076 | 0.004 | 0.747 |
| *Cyanobacteriia* | Correlation Coefficient | -0.371 | 0.007 | -.756^**^ | 0.371 | -.867^**^ | 0.350 | -.689^**^ |
|  | Sig. | 0.173 | 0.980 | 0.001 | 0.173 | 0.000 | 0.201 | 0.004 |
| *Halobacteria* | Correlation Coefficient | -0.461 | -0.266 | -0.317 | 0.438 | -0.367 | -0.342 | -0.298 |
|  | Sig. | 0.083 | 0.337 | 0.249 | 0.102 | 0.179 | 0.212 | 0.281 |
| *Bacteroidia* | Correlation Coefficient | -.777^**^ | -0.290 | -.571^*^ | .773^**^ | -0.486 | -.692^**^ | 0.095 |
|  | Sig. | 0.001 | 0.294 | 0.026 | 0.001 | 0.066 | 0.004 | 0.736 |
| *Fusobacteriia* | Correlation Coefficient | 0.103 | 0.035 | 0.393 | -0.089 | .603^*^ | -.534^*^ | .598^*^ |
|  | Sig. | 0.714 | 0.901 | 0.147 | 0.754 | 0.017 | 0.040 | 0.019 |
| *Actinobacteria* | Correlation Coefficient | -.885^**^ | -.568^*^ | -0.473 | .872^**^ | -.591^*^ | -.690^**^ | 0.013 |
|  | Sig. | 0.000 | 0.027 | 0.075 | 0.000 | 0.020 | 0.004 | 0.965 |
| *Deinococci* | Correlation Coefficient | -.663^**^ | -0.142 | -.651^**^ | .655^**^ | -.659^**^ | -0.342 | -0.293 |
|  | Sig. | 0.007 | 0.614 | 0.009 | 0.008 | 0.008 | 0.212 | 0.289 |
| *Nanosalinia* | Correlation Coefficient | -.532^*^ | -0.051 | -.525^*^ | .527^*^ | -0.503 | -0.295 | -0.325 |
|  | Sig. | 0.041 | 0.858 | 0.044 | 0.043 | 0.056 | 0.286 | 0.237 |
| ** Correlation is very significant at the 0.01 level. | | | | | | | | |
| * Correlation is significant at the 0.05 level. | | | | | | | | |
